# Compellingly negative: Bayesian analysis shows handedness and dementia are not associated

**DOI:** 10.1093/braincomms/fcad162

**Published:** 2023-05-19

**Authors:** I C McManus

**Affiliations:** Research Department of Clinical, Educational and Health Psychology, University College, London Faculty of Brain Sciences, Gower Street London, London WC1E 6BT, UK

## Abstract

This scientific commentary refers to “The role of hand preference in cognition and neuropsychiatric symptoms in neurodegenerative diseases” by Saari & Vuoksimaa (https://doi.org/10.1093/braincomms/fcad137).

**This scientific commentary refers to ‘The role of hand preference in cognition and neuropsychiatric symptoms in neurodegenerative diseases’ by Saari and Vuoksimaa (https://doi.org/10.1093/braincomms/fcad137).**

Handedness, behavioural asymmetry, and brain lateralisation are of wide interest in biology and neuroscience,^1^ not least for their possible association with clinical syndromes and pathology. In a detailed and carefully carried out study, the paper in this issue of *Brain Communications* by Saari and Vuoksimaa^2^ uses the large database of the National Alzheimer’s Coordinating Center to show that, ‘overall, handedness had no effects on most neuropsychological tests and none on neuropsychiatric symptoms’. More precisely, that means no *statistically significant effects*, which is not quite the same as statistical evidence for the absence of effects. To use a phrase from neurology, sometimes erroneously attributed to Hughlings Jackson,^3^ although contemporary with him (see https://quoteinvestigator.com/2019/09/17/absence/), ‘absence of evidence is not evidence of absence’.^4^ What is required is a Bayesian analysis, which can provide strong support for the null hypothesis, rather than merely a rejection of the alternative hypothesis. Here, that will be shown just for the general question of whether rates of left-handedness differ in neurodegenerative disorders.

Saari and Vuoksimaa^2^ compared 12 478 individuals who had neurodegenerative disorders with 17 670 cognitively unimpaired (CU) individuals. The abstract describes ‘small differences’, and as Table 1 shows, rates of non-right handedness (NRH; i.e. self-reported left-handedness, LH, or ambidexterity, *A*), differ between the four study groups: controls (CU: 10.10% NRH), and the three neurodegenerative groups [Alzheimer disease (AD), 8.96% NRH; Dementia with Lewy bodies (DLB), 9.89% NRH; and behavioural variant frontotemporal dementia (bvFTD), 10.78% NRH]. A key question, particularly given the large sample size, is whether such differences are meaningful, particularly in the Bayesian sense of asking whether the evidence may be *in favour of the null hypothesis of no differences between the groups*, a question that cannot be answered with conventional, frequentist statistics of the sort used by Saari and Vuoksimaa.^2^

**Table 1:** Comparison of handedness rates across the four diagnostic groups

Group	Handedness (RH versus *A* versus LH)			Handedness (RH versus NRH)		Total
	RH	*A*	LH	RH	NRH (LH + *A*)	
CU	15 885 (89.90%)	394 (2.23%)	1391 (7.29%)	15 885 (89.90%)	1785 (10.10%)	17 670
AD	9749 (91.04%)	179 (1.67%)	781 (7.29%)	9749 (91.04%)	960 (8.96%)	10 709
DLB	574 (90.11%)	6 (0.94%)	57 (8.94%)	574 (90.11%)	63 (9.89%)	637
bvFTD	1010 (89.22%)	14 (1.24%)	108 (9.54%)	1010 (89.22%)	122 (10.78%)	1132
Total	27 218 (90.28%)	593 (1.97%)	2.337 (7.75%)	27 218 (90.28%)	2930 (9.72%)	30 148

Comparing RH and NRH rates across the groups, a conventional chi-square test gives χ^2^ = 11.368, 3 df, *P* = 0.0099, which seems to suggest there may be a significant difference, and that the null hypothesis, H_0_, of no differences between the groups should be rejected in favour of the alternative hypothesis, H_1_. There are problems though. Large sample sizes with conventional tests over-estimate evidence against the null hypothesis^5^ and are almost certain to become significant, albeit with very small effect sizes, as sample sizes grow.^6,7^ More crucially, conventional tests can provide no evaluation of whether the data provide more support for the null hypothesis or the alternative hypothesis.

The solution is a Bayesian analysis, with the approach going back to that of Jefferys.^8^ However, as Jamil *et al.* pithily put it, Bayesian analyses seem as though they can, ‘be understood and used only by those with a high level of statistical sophistication, a fetish for archaic notation, and a desire for programming and debugging’.^5^ As a result, Bayesian methods are often not used by scientists, with software being hard to find and hard to use. A practical solution is the package *JASP* (https://jasp-stats.org/), where *JASP* stands for ‘Jeffreys’s Amazing Statistics Program’, in which a simple spreadsheet interface provides a straightforward way of carrying out Bayesian analyses as well as conventional null-hypothesis significance tests.^9,10^ Saari and Vuoksimaa^2^ carried out their analyses in *R,* for which the function *contingencyTableBF()* in the package *BayesFactor* can carry out the same analyses as *JASP*.^5^

## So what does *JASP* say about the RH–NRH data in Table 1?

Bayesian analysis assesses the Bayes Factor (BF), an odds ratio either for the alternative hypothesis relative to the null hypothesis (BF_10_), or the odds ratio for the null hypothesis relative to the alternative hypothesis (BF_01_), each being the reciprocal of the other. Odds ratios greater than one provide support for the hypothesis being tested, whereas those less than one are opposed to it.

Comparing the rate of NRH across the four groups of Table 1, BF_10_ = 0.00066, and BF_01_ = 1524.91 (see supplementary material: *SupplementaryFile_JASPanalysis_RvsNR.jasp* for full information). The odds in favour of H_1_, of there being a difference, are very low indeed (i.e. much less than one), whereas the odds in favour of the null hypothesis being the better description are very high, it being 1524 times more likely that H_0_ better describes the data than does H_1_. For comparison with conventional significance tests, with a small sample size (perhaps 20–50) a BF of 3 (‘moderate evidence’^5^) is roughly equivalent to about *P* < 0.05, and a BF of 100 (‘extreme evidence’^5^) is broadly equivalent to *P* < 0.001. However, these relationships depend on sample size, and for the present data with its 30 000 participants, and following the method of table 9 of Raftery,^7^ more significant conventional *P*-values are needed; for a BFs of 3 (moderate evidence) needing *P* ≈ 0.002 (rather than *P* = 0.05) or for a BF of 100 (extreme evidence) of about *P* ≈ 0.00006 (rather than *P* = 0.001). On that basis, the conventional chi-square statistics found in the present study might just about provide moderate evidence for a difference. Of course, null-hypothesis significance testing cannot ask what is the evidence for no difference, which the full Bayes approach can do, and then there is extreme evidence BF >>100) in favour of the null hypothesis. Despite the marginal chi-square tests, there is far more reason to accept that there are *no differences* in NRH rates between the groups. A fundamental advantage of Bayesian analysis is that it quantifies support for the null hypothesis.

Although in the bulk of their paper, Saari and Vuoksimaa only compare RH and NRH, handedness for CU, AD, DLB, and bvFTD are compared using a RH/A/LH grouping, shown in Table 1, with a significant difference reported of ‘*P* < 0.001’, presumably using a χ^2^ test. For those data, JASP gives χ^2^ = 27.62, 6 df, for which *P* = 0.00011. The Bayesian analysis, however, gives BF_10_ = 0.00000235 and BF_01_ = 426 277. The data are once again extremely strongly in favour of the null hypothesis of no difference between groups (see SupplementaryFile_JASPanalysis_RvsAvsL.jasp for further details).

Bayesian analysis in this case provides evidence for differences being *compellingly negative*, strongly supporting the absence of any difference in rates of handedness between the groups. Interpreting negative evidence is always difficult, particularly for small studies lacking statistical power. Bayesian analysis can however say when absence of evidence (non-significance) is indeed evidence of absence, and here, it is clear that evidence for right and left-handedness being associated with neurodegenerative diagnosis is indeed compellingly negative.

## Supplementary Material

fcad162_Supplementary_Data
